# The impact of humic acid on metaldehyde adsorption onto powdered activated carbon in aqueous solution†

**DOI:** 10.1039/c8ra06802j

**Published:** 2018-12-19

**Authors:** Zhuojun Li, Yuchen Yang, Ulises Jáuregui-Haza, Zhengxiao Guo, Luiza Cintra Campos

**Affiliations:** Department of Civil, Environmental and Geomatic Engineering, University College London Gower Street London WC1E 6BT UK zhuojun.li.09@ucl.ac.uk l.campos@ucl.ac.uk +44(0)-207-679-4162; Department of Chemistry, University College London Gower Street London WC1E 6BT UK yu.yang.13@ucl.ac.uk x.guo@ucl.ac.uk; Instituto Superior de Tecnologías y Ciencias Aplicadas (InSTEC), Universidad de La Habana La Habana CP 10600 Cuba ulises.jauregui@infomed.sld.cu

## Abstract

Metaldehyde has been detected in surface water and drinking water in the UK, exceeding the EU and UK standard of 0.1 μg L^−1^. The presence of natural organic matter (NOM) is considered to affect the removal efficiency of metaldehyde using traditional treatment methods such as adsorption by granular activated carbon. This paper selected humic acid (HA) to represent NOM and investigated the single and binary adsorption systems of metaldehyde and HA by powdered activated carbon (PAC). Metaldehyde was effectively removed by PAC in both systems. Since the percentage removal of metaldehyde was only 3% lower in the binary adsorption system, HA was therefore not considered as a significant compound competing with metaldehyde for adsorption sites on PAC. An adsorption equilibrium study and kinetic study for metaldehyde in a single system suggested that the Langmuir isotherm and the pseudo-second order kinetic model were more suitable in this case than the Freundlich isotherm and the pseudo-first order kinetic model. The two models revealed that the maximum adsorption capacity (*q*_m_) of metaldehyde by PAC was 28.3 mg g^−1^ and the adsorption rate (*k*_2_) was 0.16 g mg^−1^ min^−1^. The effect of pH of metaldehyde solution was also investigated in a single system. Higher percentage removal of metaldehyde was found under alkaline conditions. In contrast to metaldehyde, HA was not effectively and efficiently removed by PAC in both systems, even with higher PAC dosages and longer contact times. Hence, the microporous and mesoporous PAC was suitable for removing metaldehyde even in the binary system.

## Abstract 2

Powdered activated carbon with abundant micropores and mesopores can effectively remove metaldehyde from aqueous solution in the presence of humic acid.

## Graphical abstract

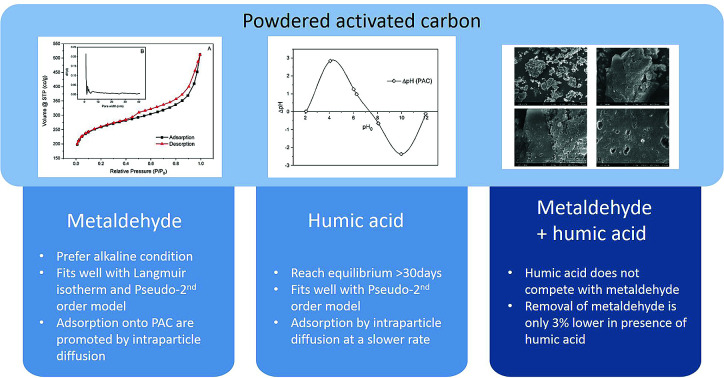

## 1. Introduction

Metaldehyde (C_8_H_16_O_4_), a highly polar organic compound and a cyclic tetramer of aldehyde (CH_3_CHO), is the active ingredient in 80% of slug repellents and it has been widely-used globally for agricultural purposes and gardening.^1^ Its IUPAC name is 2,4,6,8-tetramethyl-1,3,5,7-tetraoxocane and has a molecular mass of 176.21 g mol^−1^. The solubility of metaldehyde in water is 0.188 g L^−1^ at 20 °C.^2^ Its frequent release and persistence in natural water bodies has been polluting surface water resources, which can be used for drinking water. In fact, metaldehyde has been detected in some surface water (up to 8 μg L^−1^)^3^ and drinking water (1 μg L^−1^) which is considerably higher than the EU and UK standards of 0.1 μg L^−1^ for pesticides allowed in drinking water.^4^ Hence, it is of great significance to investigate and develop an effective treatment method to remove metaldehyde from water.

Adsorption by activated carbon and advanced oxidation processes (AOPs) are the most common treatment methods to remove pesticides in water. Granular activated carbon (GAC) is a universally-used adsorbent for treating pollutants in water for its low price and efficient results. For example, GAC filtration is quite effective in removing pharmaceutical and personal care products such as paracetamol and caffeine.^5^ However, it is not effective in removing metaldehyde in water treatment plants due to the physiochemical properties of metaldehyde^4^ such as its low log octanol/water partition coefficient (*K*_ow_) of 0.12 at 20 °C ^2^ that indicates low sorption potential.^6^ Advanced oxidation is a trending treatment method that often involves UV radiation and catalysts to break down organic pollutants into benign substances such as water and carbon dioxide. Autin *et al.* have shown that photocatalysis including UV/H_2_O_2_ and UV/TiO_2_ can degrade metaldehyde successfully, however this requires high energy input at a high cost.^7^ Therefore, a cost-effective treatment to remove metaldehyde from water is needed.

Our previous research has shown that powdered activated carbon (PAC) can be an alternative adsorbent to GAC given its excellent ability of removing metaldehyde from water with high efficiency compared to photocatalysis using nanoparticles made in the National Chemical Laboratory, India.^8^ Due to the effective results of using PAC as adsorbent, it is worth further investigating the adsorption mechanism of metaldehyde onto PAC.

Regarding adsorption processes of pollutants such as metaldehyde in water, it is necessary to take background organic materials into account. Radian and Mishael argued that interactions between pollutant and dissolved organic matter (DOM) are significant concerning the fate of pollutants in the environment and in water treatment processes.^9^ Background organic matter also affects adsorbents such as GAC and PAC. Zadaka *et al.* indicated that removal of atrazine (the most commonly-used herbicide) by GAC was reduced by 20% in the presence of DOM.^10^ Research by Matsui *et al.* showed that the adsorption capacity of PAC would be significantly affected by the presence of natural organic matter (NOM).^11^ The presence of background organic matter has negative effects on removal of metaldehyde as well. For example, Autin *et al.* claimed that NOM molecules would block the active sites of the catalyst and subsequently inhibit the degradation process of metaldehyde and the presence of background organic matter would affect the adsorption system more than oxidation.^7^ Moreover, Nabeerasool *et al.* stated that removal efficiency of metaldehyde by electrochemical processes involving novel adsorbents was reduced due to competition for active binding sites with other organic components in high NOM peat water.^12^ Hence, the background concentration of organic matter may impact the adsorption of metaldehyde onto PAC.

This study investigated the adsorption of metaldehyde onto PAC with and without the presence of background organic matter, with a control study including adsorption of only organic matter onto PAC. Specifically, humic acid (HA) was selected to represent background organic matter in this study since it is not only a significant component of background organic matter but also a common contaminant in surface water.^9^ In fact, HA accounts for 50–90% of organic matter in surface water, especially water from terrestrial origins, where the typical concentration of HA in surface water is around 30 mg L^−1^.^13^ Removal of HA in the water treatment process is also essential because the residue of HA would lead to the formation of disinfection by-products (DBPs) such as trihalomethane compounds which are carcinogenic.^14^

Therefore, this paper aimed to study closely the single and binary adsorption systems of metaldehyde and HA onto PAC which contribute to the real application of PAC in water treatment plants. Mono-component solutions containing either metaldehyde or HA were used for single adsorption system study and multi-component solutions containing metaldehyde and HA were used for binary adsorption system study. The objectives were: (1) to investigate the effect of PAC dosage, time, and pH on adsorption of metaldehyde onto PAC in single system; (2) to study the effect of PAC dosage and time on adsorption of HA onto PAC in single system; (3) to evaluate the binary adsorption of metaldehyde and HA onto PAC, including varying the concentration of HA in the binary system and adsorption time.

## 2. Materials and methods

### 2.1 Materials

Powdered activated carbon used in this study is activated charcoal, DARCO®, 100 mesh particle size powder, purchased from Sigma-Aldrich. Metaldehyde PESTANAL and humic acid sodium salt (technical grade H16752) were also obtained from Sigma-Aldrich. HPLC grade methanol and HPLC grade dichloromethane (DCM) were purchased from Fisher Scientific.

Stock solution of metaldehyde was prepared by dissolving 0.05 g metaldehyde PESTANAL in 100 mL HPLC grade methanol. Metaldehyde stock solution (100 mL of 500 mg L^−1^) can be stored between 1 and 10 °C for up to 1 year.^3^ HA stock solution was prepared by dissolving 0.5 g of humic acid sodium salt in 10 mL of NaOH (0.1 M). It was then stirred for 10 minutes before ultrapure water (MilliQ water) was added to make 500 mL of the total HA stock solution while its pH was adjusted to 7.0 by HCl (0.1 M). HA stock solution (500 mL at 1000 mg L^−1^) was then stirred again using a magnetic stirrer to ensure the HA was dissolved. After that, HA stock solution was filtered by 0.45 μm Whatman cellulose nitrate membrane filters to remove any remaining suspended solids.^13,15^ For each experiment, different amounts of metaldehyde stock solution and HA stock solution were diluted by MilliQ water to prepare corresponding sample solutions at different concentrations. The studied range of HA solution concentrations was from 3 mg L^−1^ to 90 mg L^−1^, while concentration of metaldehyde was fixed at 1 mg L^−1^, aiming to analyse the impact of different amounts of HA on removal of metaldehyde in the binary adsorption system.

### 2.2 Point of zero charge (pH_pzc_) of PAC

**Fig. 1:**
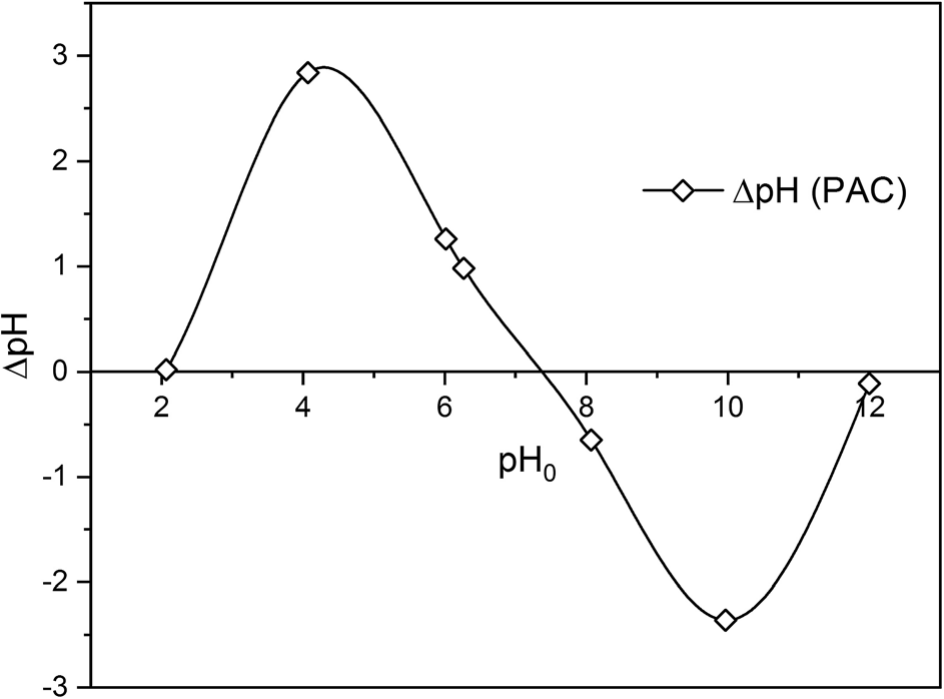
Determination of pH_pzc_ for PAC.

To study the mechanism of adsorption of metaldehyde onto PAC in single system, pH_pzc_ is an essential factor to take into account. It determines the pH value where the electrical charge density on the surface of PAC is zero and this would contribute to the understanding of the surface chemistry involving interactions between metaldehyde molecules and electrons on the surface of PAC. The method of determining pH_pzc_ of PAC follows the solid addition method.^16^ Seven 100 mL conical flasks with stoppers were filled with 50 mL of 0.1 M NaCl solution prepared from MilliQ water. For six of them, the initial pH values (pH_0_) of the solutions were adjusted using either 0.1 M HCl or 0.1 M NaOH to pH values of 2, 4, 6, 8, 10, and 12. And for one of them, pH_0_ was not adjusted and kept as the original pH of NaCl solution which was 6.27. After that, 0.06 g of PAC were added into each flask and mixed well with the NaCl solutions. These flasks were left to equilibrate for 48 hours with intermittent manual mixing. Finally, the final pH values (pH_48_) of the mixtures were recorded. The differences between pH_0_ and pH_48_ were calculated as ΔpH which were then plotted against pH_0_. Fig. 1 shows that the pH_pzc_ of PAC which is 7.35 *i.e.* the point where ΔpH = 0.

### 2.3 Adsorption experiments

To study the removal of metaldehyde from water using PAC in both systems, three sets of experiments were carried out. The percentage removal of pollutants, and the adsorbed amount of pollutants onto PAC were calculated using the following equations:^13,16^1
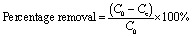
2
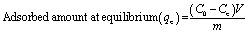
3
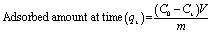


Eqn (1) describes the percentage removal of metaldehyde or HA from water where *C*_0_ is the initial concentration of adsorbate before treatment and *C*_e_ is the final concentration of adsorbate after treatment at equilibrium. Eqn (2) describes the amount of metaldehyde or HA adsorbed at equilibrium, where *V* is the volume of the solution and *m* is the mass of adsorbent. Eqn (3) is similar to eqn (2) where *q*_t_ is the amount of metaldehyde or HA adsorbed at a specific time and *C*_t_ is the concentration of adsorbate at a specific time.

All experiments were performed as batch tests using a mono-component metaldehyde solution for single system study, a mono-component HA solution for single system study, and a multi-component solution containing metaldehyde and HA for binary system study, together with added PAC and consistent mixing by magnetic stirrer to ensure PAC was in contact with the solutions. Sample solution (3 mL) was taken and used as triplicates (1 mL per triplicate) and filtered through 0.45 μm Whatman cellulose nitrate membrane to remove suspended PAC from the solution at the end of the 2 hour experiments. For single adsorption of metaldehyde, PAC dosage, pH of the solution, and adsorption time were varied. For single adsorption of HA, PAC dosage and adsorption time were varied. For the binary adsorption of metaldehyde and HA, initial HA concentration and adsorption time were varied (details of adsorption experiment are provided in ESI†).

### 2.4 Analytical methods

Metaldehyde was analysed by gas chromatography (Perkin Elmer precisely Clarus 500) with mass spectrometry (GC-MS), as recommended by the UK Environment Agency.^3^ Sample solutions containing metaldehyde were taken and analysed using the same solid phase extraction (SPE) and the GC-MS methods described in our previous research with a detection limit of metaldehyde from 1 to 5 μg L^−1^.^8^ The concentration of samples containing HA were determined by CamSpec M550 Double Beam Scanning UV-Vis Spectrophotometer at 254 nm. Sample solutions from binary adsorption tests containing both metaldehyde and HA were analysed by both methods to determine the concentrations of metaldehyde and HA separately. The presence of HA does not affect the detection of metaldehyde in the binary adsorption system and *vice versa*. pH values were measured by the pH meter SevenMulti, Mettler Toledo. Brunauer–Emmett–Teller (BET) specific surface area analysis of PAC was done by Autosorb-iQ2 automated gas sorption analyser (Quantachrome Instruments) *via* adsorption and desorption of nitrogen gas at 77 K after PAC sample being degassed at temperature of 180 °C for 24 hours. Scanning Electron Microscope (SEM) images of PAC were taken by JSM-6701F Field Emission Scanning Electron Microscope (JEOL) at 10 kV under secondary electron imaging mode (SEI).

## 3. Results and discussion

### 3.1 PAC characterization

#### 3.1.1 BET analysis

**Fig. 2:**
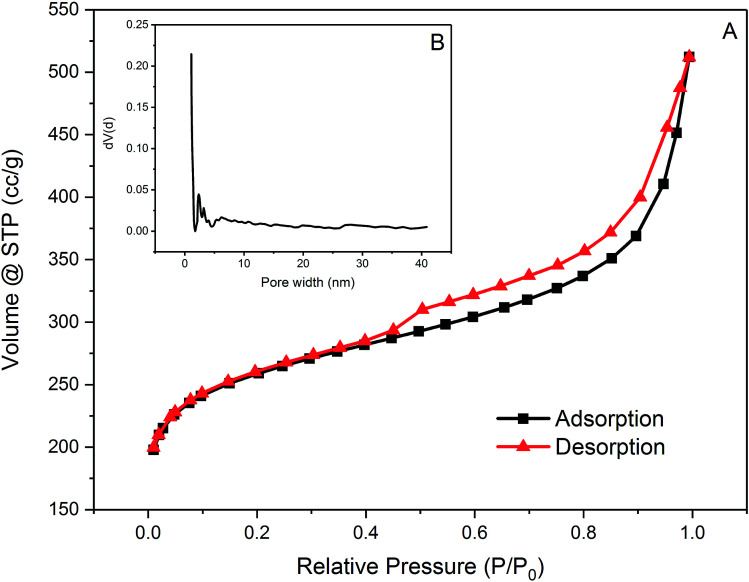
(A) Nitrogen adsorption and desorption isotherm of PAC at 77 K and (B) pore distribution of PAC.

Fig. 2(A) shows the 77 K nitrogen adsorption and desorption isotherm of PAC. The BET specific surface area of PAC was determined to be 962 m^2^ g^−1^ using 5 points selected from relative pressure (*P*/*P*_0_) ranged from 0.02 to 0.1, with a total pore volume of 0.792129 cm^3^ g^−1^ determined at 0.99388 relative pressure (*P*/*P*_0_). The isotherm exhibited a combination of type I and IV isotherms with hysteresis loops at relative pressure above 0.4, which indicates the combination of both micro- and mesopores. This phenomenon can be further evidenced by the pore size distribution analysis using Density Functional Theory (DFT) methods, as shown in Fig. 2(B).

PAC is dominated by micropores which have pore widths smaller than 2 nm with abundant mesopores with pore widths between 2 nm and 5 nm. Regarding its pore size distribution, PAC is considered to be favourable for adsorption of small molecules such as metaldehyde for its large numbers of micro-and mesopores. However, for compounds which has a large and complex structure, such as HA, adsorption onto this PAC might not be as effective.

#### 3.1.2 SEM analysis

**Fig. 3:**
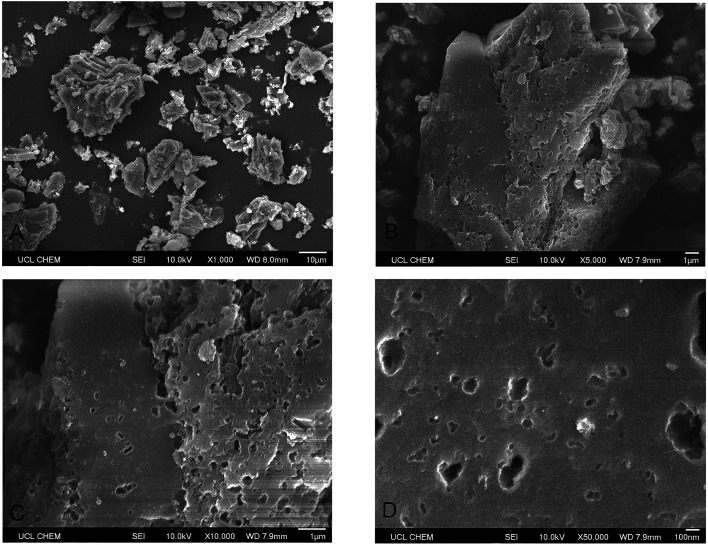
SEM images of PAC on different scales. (A) (× 1000 magnification): an overview of PAC grains scattering around; (B) (× 5000 magnification): PAC grain with visible pores on surface; (C) (× 10 000 magnification): the edges and surface of PAC grains which are potential adsorption sites; (D) (× 50 000 magnification): macropores can be seen on the surface with scale bar of 100 nm.

Fig. 3 illustrates the SEM images of PAC which shows its structure and surface morphology. PAC grains are scattered around and their sizes vary from a few microns to 20 μm. The edges of the grains are angular while the surface is rough and porous. Visible pores can be seen on both edges and surface of PAC grains.

### 3.2 Removal of metaldehyde in single adsorption system

#### 3.2.1 Effect of PAC dosage

**Fig. 4:**
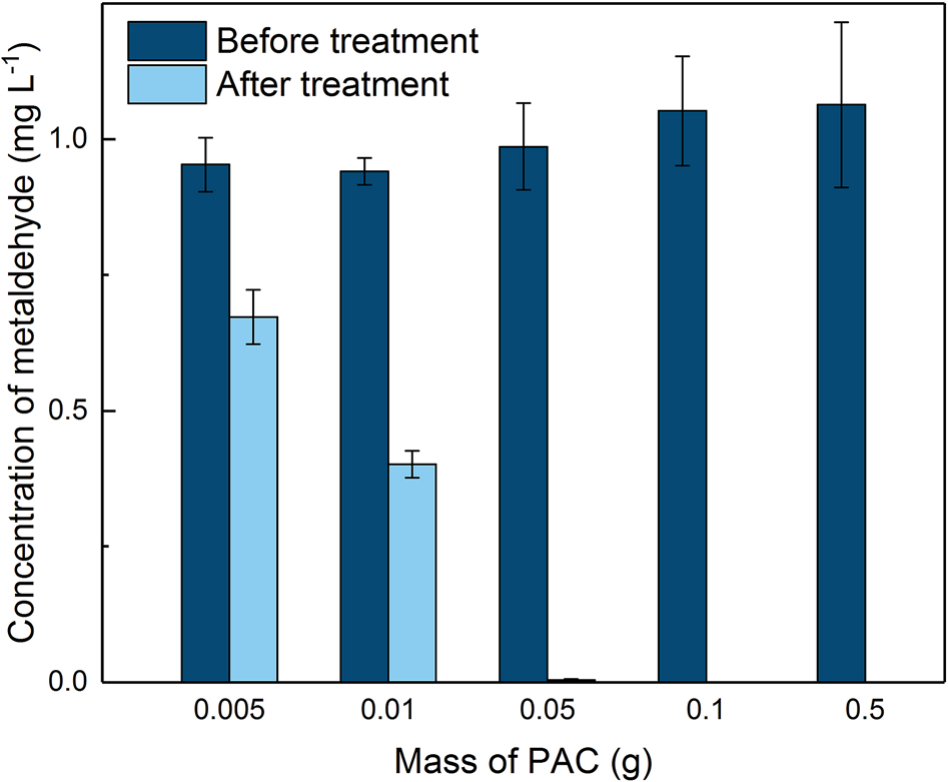
Concentration of metaldehyde before and after 2 hour treatment using different PAC dosage in single adsorption system.

Fig. 4 shows concentrations of metaldehyde before and after treatment without adjusting the pH of metaldehyde solution in the single system. It can be seen that metaldehyde was effectively removed, especially with higher PAC dosages. An ANOVA single-factor statistic test confirmed that there were significant differences (*p* < 0.05) between concentrations of metaldehyde before and after 2 hour PAC treatment. Percentage removal of metaldehyde increased from 30.3% to 99.6% when PAC dosage increased from 0.005 g to 0.05 g. When PAC dosage was higher than 0.05 g, metaldehyde could not be detected after treatment, suggesting that its concentration was below the detection limit of GC-MS for metaldehyde (1 to 5 μg L^−1^).^8^

#### 3.2.2 Effect of adsorption contact time

**Fig. 5:**
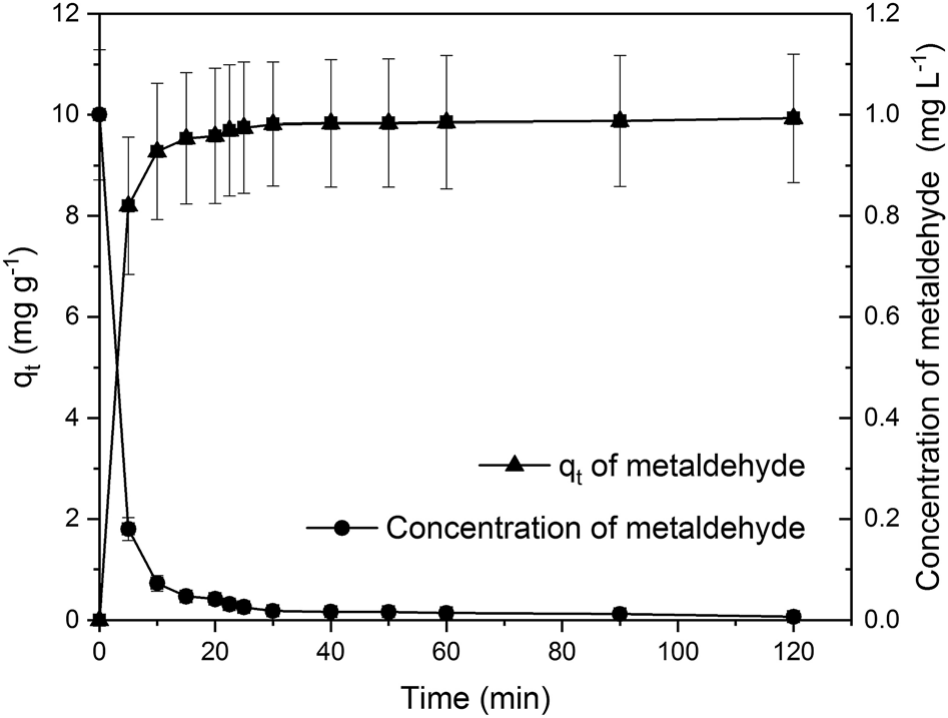
Effect of time on metaldehyde removal by PAC in single adsorption system.

Fig. 5 shows the amount of metaldehyde adsorbed onto PAC (*q*_t_) over time. It can be seen that metaldehyde was rapidly adsorbed onto PAC in the first 5 to 10 minutes and gradually plateaued from 30 minutes, reaching equilibrium (*q*_e_ = 9.932 mg g^−1^) with 99.3% removal of metaldehyde which behaved the same as that shown in our previous research.^8^

#### 3.2.3 Effect of pH of metaldehyde solution

**Fig. 6:**
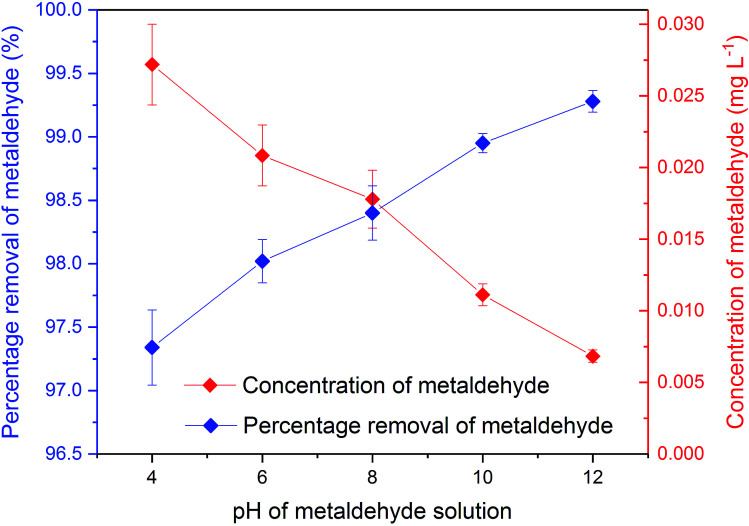
Concentration and percentage removal of 500 mL of 1 mg L^−1^ metaldehyde after 2 hour treatment using 0.05 g of PAC under different pH environments in single adsorption system.

Fig. 6 presents the removal of metaldehyde under different pH conditions. Metaldehyde was effectively removed by PAC over the pH values tested. It is noted that under very acidic conditions such as pH 2, metaldehyde will undergo hydrolysis and decompose into acetaldehyde.^17^ This was confirmed that 1 mg L^−1^ prepared metaldehyde solution became 0.2 mg L^−1^ at pH 2 without any treatment. For this reason, this study was done starting at pH 4.

There were significant differences (*p* < 0.05) between concentrations of metaldehyde before and after 0.05 g dosages of PAC treatment at pH 4, 6, 8, 10, and 12. Removal of metaldehyde slightly increased from 97.4% to 99.3% as the pH increased from 4 to 12. This suggests that adsorption of metaldehyde onto PAC is favoured in an alkaline environment, which can be confirmed by the pH_pzc_ of PAC at 7.35. The surface of PAC is negatively charged when the pH is higher than 7.35 and will interact with positively charged species and *vice versa*.^18^ Metaldehyde as a highly polar chemical with a positively charged surface will therefore prefer the negatively charged surface of PAC under a high pH environment.

#### 3.2.4 Adsorption kinetic studies of metaldehyde in single adsorption system

Experimental data from Section 3.2.2 were analysed using the two most commonly used kinetic models; the pseudo-first order and the pseudo-second order models.

The pseudo-first order model was proposed by Lagergren for a liquid–solid adsorption system which is based on solid capacity.^19,20^ It assumes that the adsorption rate is proportional to the difference of *q*_t_ and *q*_e_, demonstrated by eqn (4) and (5) where *k*_1_ is the pseudo-first order kinetic rate constant.4
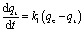

5 ln(*q*_e_ − *q*_t_) = ln *q*_e_ − *k*_1_*t*

Experimental data were not well fitted to the pseudo-first order model with *R*^2^ = 0.6532 (Fig. 1 in ESI†). The calculated value of *q*_e_ from this model was −2.527 mg g^−1^ and *k*_1_ was 0.0203 min^−1^. Although the negative value of *q*_e_ clearly contradicted with the experimental value of *q*_e_ = 9.932 mg g^−1^, the value of *k*_1_ suggests quite a fast adsorption rate. It is much higher than the *k*_1_ of 7.5 × 10^−3^ min^−1^ for adsorption of metaldehyde onto GAC stated by Salvestrini *et al.*^21^ This implies that the abundant mesopores in PAC facilitate the fast diffusion of metaldehyde molecules into micropores while GAC does not have large number of mesopores and micropores which assist the efficient diffusion.^22^

The pseudo-first order model can also be separated into two gradient stages from 0 minute to 30 minutes and from 30 minutes to 120 minutes which corresponds to fast adsorption and slow adsorption, respectively (Fig. 2 in ESI†). Data were better fitted with two stages (*R*_0–30 min_^2^ = 0.9031, *R*_30–120 min_^2^ = 0.9882). According to Li *et al.*, for a chemically-controlled model, two gradients suggest two chemically different adsorption sites.^23^ For a diffusion-controlled model, two different rates imply different diffusion rates. The first rate (*k*_1_ = 0.0571 min^−1^) indicates a higher rate of diffusion *via* the easily accessed external adsorption sites and macropores. The second rate (*k*_1_ = 0.0053 min^−1^) which is much slower than the first one represents slower rate of diffusion *via* mesopores and micropores. For this model, they stated that the adsorption rate is determined by the pore diffusion rate.^23^ In this study, it is unlikely that PAC has two chemically different adsorption sites. Therefore, the adsorption of metaldehyde fitted into the pseudo-first order model can be best explained as diffusion-controlled.

The pseudo-second order equation^24^ describes the adsorption rates as proportional to the difference of *q*_e_ and *q*_t_ squared as shown by eqn (6) and (7) where *k*_2_ is the pseudo-second order kinetic rate constant.^21^ The pseudo-second order model assumes that the adsorption rate could be explained by the intraparticle diffusion model. It is limited by the rate of adsorbate diffusion inside the pores of adsorbent, and *k*_2_ is dependent on the initial adsorbate concentration and solid-solution ratio.^25^6
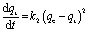
7
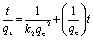


Data were very well fitted to the pseudo-second model with *R*^2^ = 0.9999 (Fig. 3 in ESI†). Calculated *q*_e_ from this model is 9.97 mg g^−1^ which is very close to the experimental value of 9.932 mg g^−1^. This confirms that the pseudo-second model is suitable for analysing these data, indicating that the intraparticle diffusion mechanism could probably dominate the adsorption process of metaldehyde onto the PAC in the study and the rate of direct adsorption which is regarded as surface reaction controls the adsorption kinetics.^26^ The value of *k*_2_ in this study is 0.16 g mg^−1^ min^−1^ which is much higher than the one obtained by Salvestrini *et al.*^21^ (8 × 10^−5^ g mg^−1^ min^−1^) using GAC, implying very fast adsorption rate of metaldehyde onto PAC.

#### 3.2.5 Adsorption isotherm studies of metaldehyde in single adsorption system

**Fig. 7:**
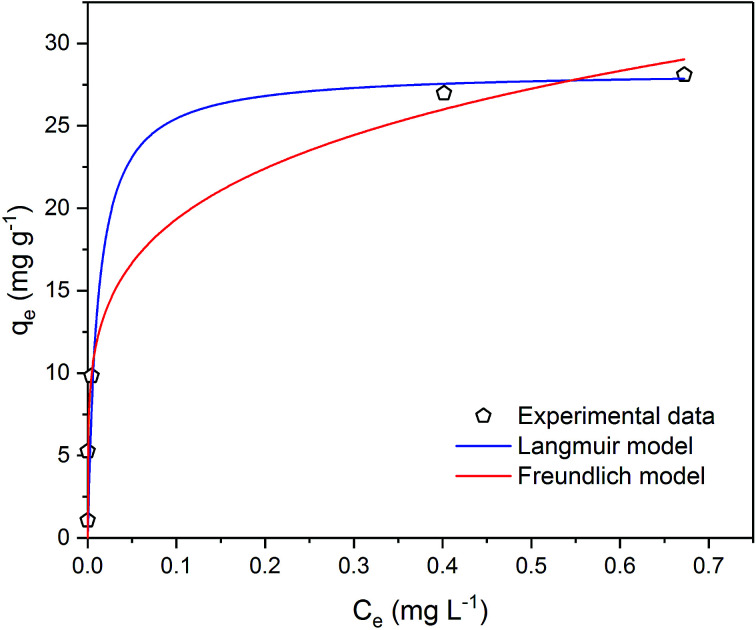
Metaldehyde adsorption equilibrium curve with Langmuir and Freundlich isotherm models fitted to experimental data in single adsorption system.

Taking into account the results of adsorption kinetics, the adsorption isotherm for metaldehyde was determined at 2 hours. Fig. 7 shows the single adsorption equilibrium curve of metaldehyde at 25 °C.

It is very important to select the best fitted isotherm model to correlate the equilibrium curve shown in Fig. 7. Freundlich isotherm is generally used for heterogeneous adsorption systems. It predicts that the adsorbate concentrations on the adsorbent will increase given there is an increase of the adsorbate in the liquid. Eqn (8) and (9) describe the Freundlich isotherm model where 1/*n* is the heterogeneity factor (*i.e.* adsorption intensity) and *K*_F_ is the Freundlich constant (*i.e.* adsorption capacity).^16,19^

8 *q*_e_ = *K*_F_*C*_e_^1/*n*^

9
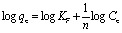


Data were well fitted with *R*^2^ = 0.9966 (Fig. 4 in ESI†) and the fitting gave 1/*n* value of 0.211 (*n* = 4.732) and the *K*_F_ value of 0.176 (mg g^−1^)/(mg L^−1^)^1/*n*^. As Kumar *et al.* stated that 1/*n* indicates the relative distribution of energy sites and the higher the 1/*n*, the higher the affinity is between adsorbate and adsorbent, and the adsorbent sites will be more heterogeneous.^16^ In this case, 21.1% of the active adsorption sites would have equal energy levels. A low value of 1/*n* such as 0.211 suggests that the affinity between the PAC used in our study and metaldehyde is low and the heterogeneity of PAC sites is low. As an indicator of adsorption capacity, the *K*_F_ value obtained in this study is 0.176 (mg g^−1^)/(mg L^−1^)^1/*n*^, more than 10 times smaller than the one obtained by Kumar *et al.* around 2.5 (mg g^−1^)/(mg L^−1^)^1/*n*^.^16^ Kumar *et al.* argued that their high *K*_F_ value suggests effective adsorption;^16^ therefore, the low *K*_F_ value obtained in our study cannot explain the effective removal of metaldehyde by PAC in the experiment. The low 1/*n* value also suggests that the heterogeneity of the system is low, implying that Freundlich isotherm is not suitable for fitting the data.^16^

Langmuir isotherm is a commonly used model for adsorption studies with homogeneous surfaces. It assumes the existence of monolayer coverage of the adsorbate at the surface of the adsorbent. Therefore, the adsorbent has a maximum capacity for the adsorbate and once a saturation is reached, there will be no more adsorption.^19^Eqn (10) and (11) describe Langmuir isotherm where *K*_L_ (L mg^−1^) is the Langmuir constant, and *q*_m_ (mg g^−1^) is the saturation/maximum adsorption capacity.10
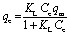
11
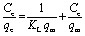


Data were very well fitted with *R*^2^ = 0.9994 (Fig. 5 in ESI†) and the fitting gave *q*_m_ of 28.3 mg g^−1^ and *K*_L_ of 88.3 L mg^−1^. The maximum adsorption capacity *q*_m_ represents the saturation of the one molecule thick metaldehyde on the surface of PAC at equilibrium. *K*_L_ correlates to the concentration where the amount of metaldehyde adsorbed onto PAC is equal to *q*_m_/2. A high *K*_L_ value in this case indicates high affinity of metaldehyde to bind with PAC which can be confirmed by the effective removal of metaldehyde. Therefore, Langmuir isotherm is suitable for representing metaldehyde adsorption onto PAC.

Statistically, both Freundlich and Langmuir models were fitted to the experimental data using a non-linear regression algorithm^27^ which selects the best-fitting model based on the experimental data. The Akaike information criterion (AIC) method finds the best model considering the residual sum of squares (RSS) and the number of free parameters.^28^ In this study, AIC analysis confirmed that the Langmuir model is more suitable since it has a lower AIC value of 11.5 compared to the Freundlich model which has an AIC value of 13.1.

### 3.3 Removal of HA in single adsorption system

#### 3.3.1 Effect of PAC dosage

**Fig. 8:**
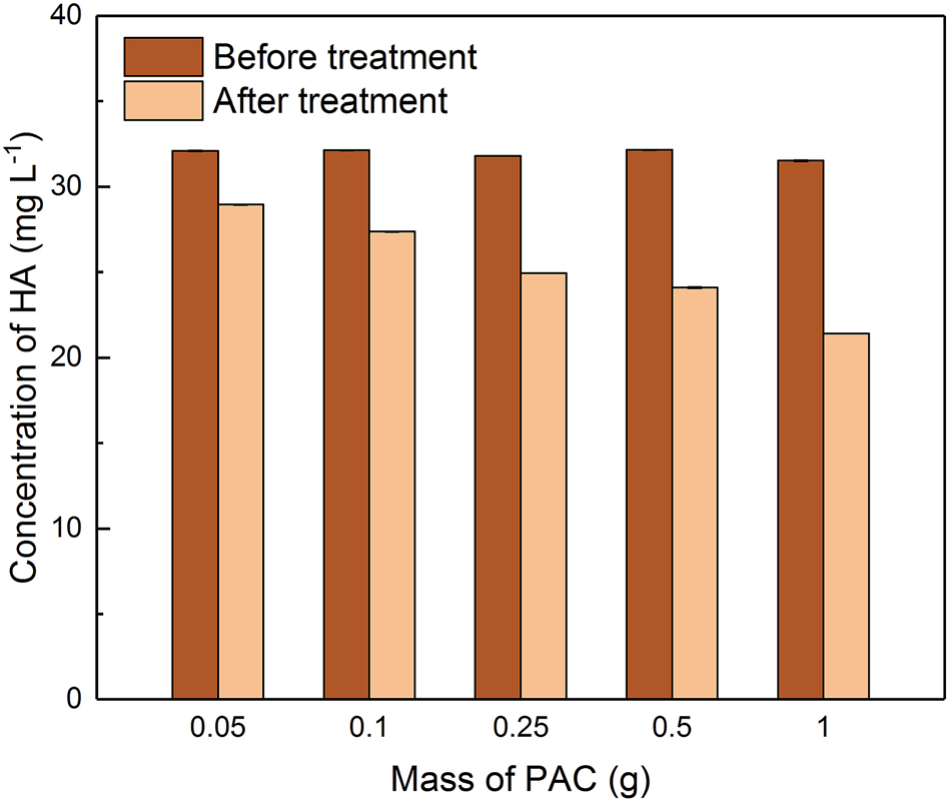
Concentration of HA before and after 2 hour treatment using different PAC dosages in single adsorption system.

Fig. 8 shows the concentrations of HA before and after different dosages of PAC treatment. HA was moderately removed by adsorption on PAC. The percentage removal of HA ranged from 9.8% to 32% with increasing PAC dosage. Although the initial concentration of HA before treatment (*C*_0_ = 30 mg L^−1^) was much higher than that of metaldehyde (*C*_0_ = 1 mg L^−1^), a high dosage of PAC (*e.g.* 1 g) could still only remove 32% of HA in 2 hours.

#### 3.3.2 Effect of adsorption contact time

**Fig. 9:**
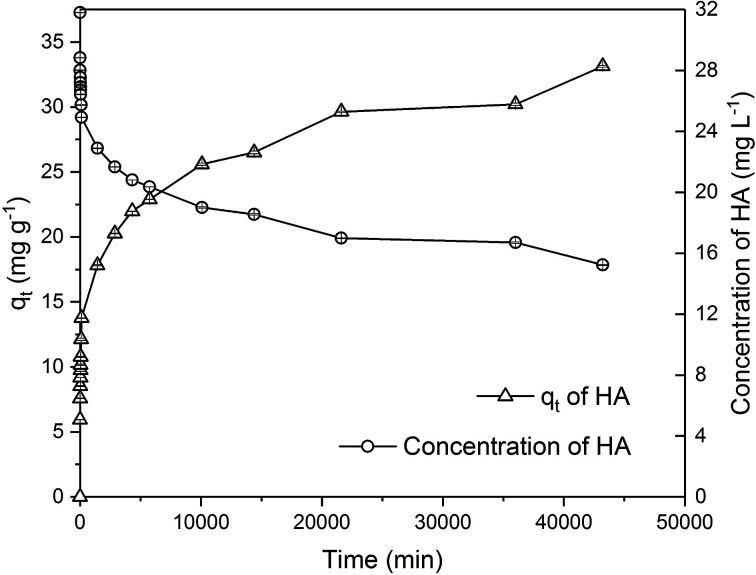
Effect of time on HA removal by PAC in single adsorption system.

A dose of 0.25 g of PAC was chosen in this experiment to remove HA (*C*_0_ = 30 mg L^−1^) from 500 mL water with the aim of identifying the time required to reach equilibrium. Fig. 9 shows the adsorption of HA from 0 to 30 days and it suggests that there is no clear sign of adsorption of HA that would gradually plateau and reach equilibrium. At the end of the 30 day experiment, 50% of HA was removed. Based on this trend, it is highly possible that HA will continue to be removed even after the 30 day treatment.

**Fig. 10:**
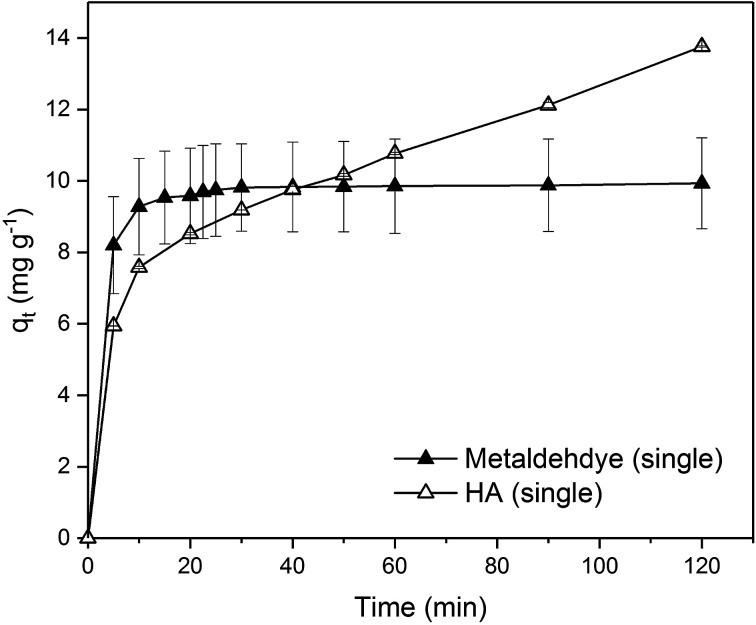
Comparison of adsorption of metaldehyde and HA by PAC in 2 hours in single adsorption system.

Due to the different behaviour of adsorption of HA and metaldehyde onto PAC over time, the first 120 minutes of HA adsorption curve was compared with that of metaldehyde in single adsorption system (Fig. 10). In the first 5 minutes, both HA and metaldehyde were rapidly adsorbed on PAC. However, after that, adsorption of metaldehyde slowed down significantly and trended towards equilibrium while adsorption of HA was not as fast over the first 5 minutes but kept increasing at a steady, slightly slower rate. Interestingly, the trend of the adsorption of HA onto PAC over the shorter time scale (from 0 minute to 120 minutes) was very similar to the trend over the longer time scale (from 0 minutes to 30 days).

#### 3.3.3 Adsorption kinetic study of HA in single adsorption system

From Fig. 9 in Section 3.3.2, it was found that the adsorption of HA by PAC did not reach equilibrium in 30 days. Therefore, Langmuir and Freundlich adsorption isotherm models and the pseudo-first order kinetic model cannot be applied to the data since these models require the value of *C*_e_ and *q*_e_ when the system reaches equilibrium. However, data from Section 3.3.2 could be applied to the pseudo-second order kinetic model which only requires the value of *q*_t_.

Data were well fitted to the pseudo-second order kinetic model with *R*^2^ = 0.9918 (Fig. 6 in ESI†), suggesting that adsorption of HA onto the PAC used in this study could also be explained by the intraparticle diffusion model. Calculated *q*_e_ is 31.65 mg g^−1^ while *q*_t_ at the end of 30 days was found to be 33.14 mg g^−1^ which implies the system would have reached equilibrium with a *q*_e_ of 31.65 mg g^−1^ in 30 days if the system followed the pseudo-second order model completely. The value of *k*_2_ is 4.23 × 10^−5^ g mg^−1^ min^−1^, indicating a very slow adsorption rate compared to that of metaldehyde.

To compare with other studies, it was argued by Capasso *et al.* that there was fast adsorption of HA onto zeolitic tuffs at first, then it reached pseudo steady-state in a few days; however, uptake of HA increased again and reached equilibrium in 2 months.^29^ The trend reported by them and the trend of HA adsorbed onto the PAC in this study share some similarities. Fig. 9 demonstrates that there was 29% removal of HA in the first day, and 50% removal of HA at 30 days in this study while Capasso *et al.* found 50% removal of HA on the first day and 96% removal of HA at the end of their experiment. They explained this two-step behaviour by the fact adsorption of HA has two routes and one of them occurs over a few days and another is relatively slower and occurs over a longer time period.^29^ This also seemed very similar to the diffusion controlled model of adsorption discussed in Section 3.2.4 that had two adsorption rates.

Moreover, Kołodziej *et al.* used modified activated carbons with different pH_pzc_ for HA adsorption and suggested that adsorption of HA seems to favour adsorbents with relatively low or neutral pH_pzc_.^30^ In terms of kinetic analysis, Kołodziej *et al.* found *q*_e_ was 32.89 mg g^−1^ for adsorption of brown HA onto hydrogen treated activated carbon using the pseudo-second order model which was very similar to 31.65 mg g^−1^ obtained in this study. However, their *k*_2_ value was 9.57 × 10^−3^ g mg^−1^ min^−1^, much higher than the *k*_2_ obtained in this study because adsorption of HA reached equilibrium in a shorter time in their study.^30^

### 3.4 Removal of metaldehyde and HA in binary adsorption system (competitive adsorption)

#### 3.4.1 Effect of the initial concentration of HA

**Fig. 11:**
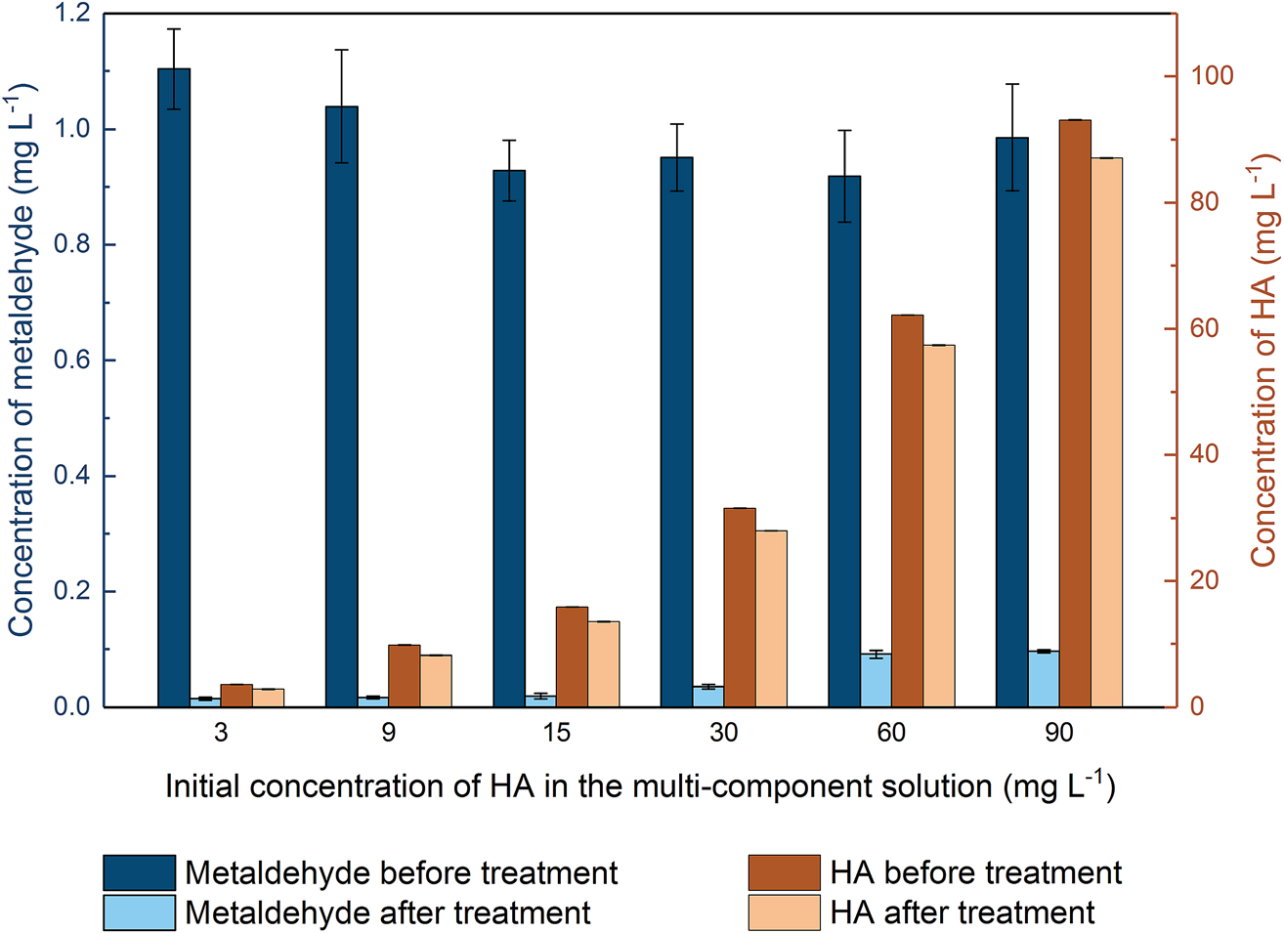
Concentration of metaldehyde and HA in the binary adsorption system before and after PAC treatment, with different initial concentrations of HA while initial concentrations of metaldehyde were fixed to be 1 mg L^−1^.

Fig. 11 shows that metaldehyde was effectively removed in the binary system with different initial HA concentrations (*p* < 0.05) after PAC treatment (competitive adsorption). There was 90.2% removal of metaldehyde even with a very high concentration of HA present (90 mg L^−1^). This finding suggests that the presence of HA does not significantly affect the removal of metaldehyde by PAC in the binary system. The removal percentage of metaldehyde decreased from 98.6% to 90.2% with the increase of concentration of HA from 3 mg L^−1^ to 90 mg L^−1^. It also indicates that HA were moderately removed from the binary system (*p* < 0.05). Percentage removal of HA decreased from 20.5% at *C*_0_ = 3 mg L^−1^ to 6.5% at *C*_0_ = 90 mg L^−1^.

#### 3.4.2 Effect of adsorption contact time

**Fig. 12:**
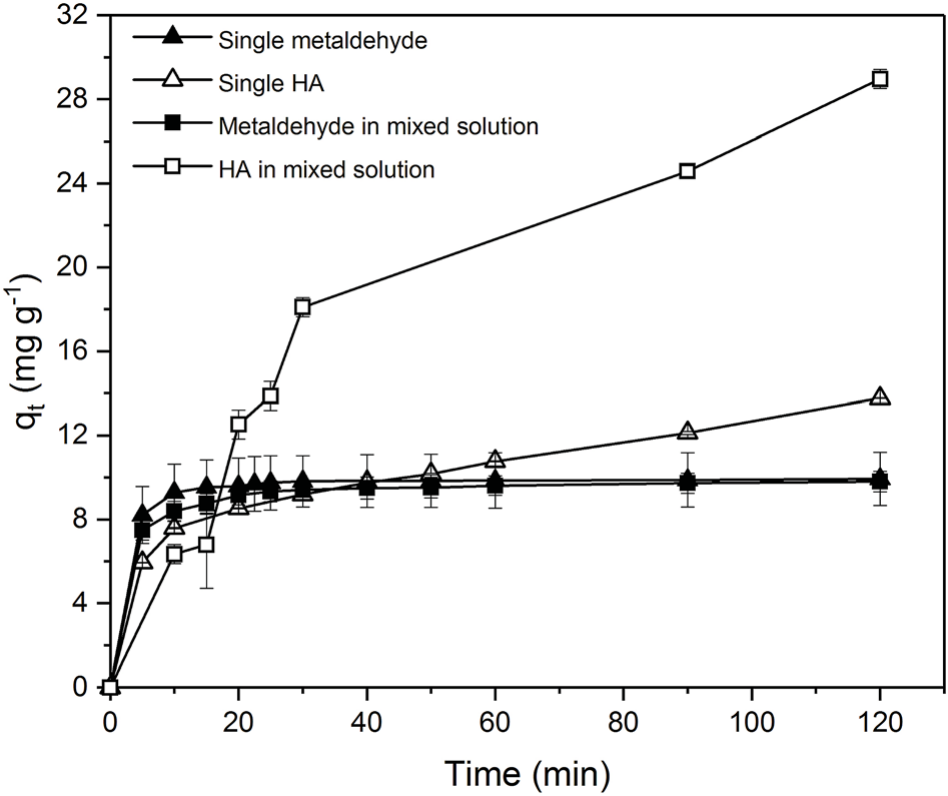
Effect of time on single adsorption of metaldehyde (1 mg L^−1^) with 0.05 g PAC, single adsorption of HA (30 mg L^−1^) with 0.25 g PAC, and binary adsorption of metaldehyde (1 mg L^−1^) and HA (30 mg L^−1^) with 0.05 g PAC.

Fig. 12 compares the single adsorption of metaldehyde (1 mg L^−1^) using 0.05 g PAC; single adsorption of HA (30 mg L^−1^) using 0.25 g PAC; and binary adsorption of metaldehyde (1 mg L^−1^) and HA (30 mg L^−1^) using 0.05 g PAC. Adsorption of metaldehyde in the binary system was only slightly lower and slower than that of the single system but they both showed the same trend of fast adsorption in the first 5 minutes then slow adsorption which led to equilibrium in approximately 2 hours with 98% removal of metaldehyde. In contrast, *q*_t_ of HA in the binary system was larger than that of the single system because a different PAC dosage was used since 0.05 g PAC could effectively remove 1 mg L^−1^ of metaldehyde but not 30 mg L^−1^ HA in the binary system. Therefore 0.25 g PAC was used for single adsorption of HA (30 mg L^−1^) to investigate whether PAC could effectively remove HA and reach equilibrium. In fact, 21.7% of HA was removed by 0.25 g PAC in the single system while 9.1% of HA in the binary system was removed by 0.05 g PAC. Although the mass of PAC in the single system was 5 times higher than that of the binary system, removal of HA in the single system is approximately two times higher than the binary system which suggests that removal of HA did not significantly benefit from the high PAC dosage. However, the adsorption trend of HA in both systems were quite similar. They both showed fast adsorption at the beginning and kept increasing at a relatively steady rate.

#### 3.4.3 Adsorption kinetic studies for metaldehyde and HA in binary adsorption system

The pseudo-first order and the pseudo-second order kinetic models were applied to adsorption of metaldehyde onto PAC in the binary system (Fig. 7 and 8 in ESI†). Data were not very well fitted to the pseudo-first order model (*R*^2^ = 0.8192). Calculated *q*_e_ was −0.9276 mg g^−1^ and *k*_1_ was 0.0193 min^−1^. *k*_1_ value was slightly lower than the one obtained for metaldehyde in single system, due to the presence of HA. On the other hand, data were well fitted to the pseudo-second order model (*R*^2^ = 0.9998) with a calculated *q*_e_ of 9.88 mg g^−1^ which is very close to the experimental value of 9.8 mg g^−1^. *k*_2_ was 0.069 g mg^−1^ min^−1^ which is less than half of the *k*_2_ obtained for metaldehyde in single system. This confirms that adsorption of metaldehyde required a longer time in binary system.

The pseudo-second order model was applied to adsorption of HA in the binary system (Fig. 9 in ESI†) with *R*^2^ = 0.8459. Calculated *q*_e_ is 35.71 mg g^−1^ which is very close to the *q*_e_ obtained for adsorption of HA onto PAC in single system and *k*_2_ is 7.84 × 10^−4^ g mg^−1^ min^−1^ which means adsorption of HA in the binary system is faster than that of the single system, implying the presence of metaldehyde would promote the adsorption of HA.

### 3.5 Adsorption mechanism of metaldehyde and HA

In general, the PAC used in this study as an adsorbent was very effective to remove metaldehyde from water, especially in the single adsorption system (Table 1 in ESI†). Combined with the BET analysis of PAC, effective removal of metaldehyde could be explained by the characteristics of the PAC used in this study. The specific surface area of the PAC is quite large which is 962 m^2^ g^−1^ and it is dominated by micropores with abundant mesopores present. According to Busquets *et al.*, adsorption of metaldehyde could be greatly enhanced with carbon materials that are highly microporous with the presence of mesopores which could assist diffusive transport.^22^

**Table 1:** Adsorption kinetic analysed for metaldehyde and HA

	Kinetic constants	
	The pseudo-first order constant *k*_1_ (min^−1^)	The pseudo-second order constant *k*_2_ (g mg^−1^ min^−1^)
Metaldehyde (single)	0.0203	0.16
HA (single)	n/a	4.23 × 10^−5^
Metaldehyde (binary)	0.0193	0.069
HA (binary)	n/a	7.84 × 10^−4^

In this study, the average removal of metaldehyde in the binary system of metaldehyde (1 mg L^−1^) and HA (30 mg L^−1^) was around 97.5% by 100 mg L^−1^ PAC while removal of 25 mg L^−1^ metaldehyde in surface water and tap water is 94% by Nguyen *et al.*^31^ with the oxidation reaction using 100 mg L^−1^ graphene oxide and 1% H_2_O_2_*via* modified Fenton's process. In both researches, the presence of HA only slightly affects the removal of metaldehyde. Nguyen *et al.*^31^ argued that this is due to the limited adsorption capacity of graphene oxide for DOM or the oxidation process takes place very quickly before the active sites of graphene oxide become occupied. In our study, compared to metaldehyde, HA was not effectively removed by the PAC used. Moreover, when increasing the proportion of HA in the binary system, the removal of metaldehyde was still only moderately affected. This could be explained by the pore structure of the PAC used. Micropores and mesopores are suitable for adsorbing small-sized compounds with a stable structure such as metaldehyde. On the other hand, HA is a large and complex compound with a variety of components which could not fit in the micropores of this PAC. The average 10–20% removal of HA could be explained by the attachment of HA to the surface and limited macrospores of PAC. Hence, regarding the removal of metaldehyde by the PAC used, HA is not considered as a competitive compound.

Table 1 demonstrates the kinetic constants analysed for metaldehyde and HA in both systems, and Table 2 shows the equilibrium study of metaldehyde in single adsorption system. A table of comparing adsorption capacities for metaldehyde, specific surface area, and adsorption efficiency of different adsorbents can be found in our previous research.^8^ In this study, the maximum adsorption capacity of PAC for metaldehyde was 28 mg g^−1^ which is much higher than the 15 mg g^−1^ of GAC used by Busquets *et al.*^22^ And the adsorption rate (*k*_2_) of 0.16 g mg^−1^ min^−1^ for metaldehyde in single system was much higher than 8 × 10^−5^ g mg^−1^ min^−1^ of GAC used by Salvestrini *et al.*,^21^ and 5.8 × 10^−4^ g mg^−1^ min^−1^ of GAC used by Tao and Fletcher^32^ which suggests that single adsorption of metaldehyde onto PAC is very fast. GAC has also been used in this study for adsorption of metaldehyde and HA to compare with PAC. It was found that the dosage of GAC required to remove the same amount of metaldehyde in single system was 10 times larger than PAC. Details of comparison are shown in Table 2 in the ESI.†

**Table 2:** Adsorption equilibrium study for metaldehyde in single system

	Equilibrium isotherm constants			
	Langmuir isotherm		Freundlich isotherm	
	*K*_L_ (L mg^−1^)	*q*_m_ (mg g^−1^)	*K*_F_ (mg g^−1^)/(mg L^−1^)^1/*n*^	1/*n*
Parameter values	88.3	28.3	0.176	0.211
Residual sum of square (RSS)	33.6		30.6	
Akaike information criterion (AIC)	11.5		13.1	

Additionally, *k*_2_ decreased to 0.069 g mg^−1^ min^−1^ for metaldehyde in binary system, indicating HA moderately affected the adsorption rate of metaldehyde in binary system and led to a delay for the system to reach equilibrium. Adsorption rates of HA were much slower than metaldehyde in both systems. However, it is interesting that the adsorption rate of HA in binary system (7.84 × 10^−4^ g mg^−1^ min^−1^) was higher than that of the single system (4.23 × 10^−5^ g mg^−1^ min^−1^). This suggests that in the binary system, metaldehyde promotes the adsorption rate of HA while HA would slow down the adsorption rate of metaldehyde.

## 4. Conclusion

Metaldehyde could be effectively removed from aqueous media by the PAC used in this study with a maximum adsorption capacity (*q*_m_) of 28.3 mg g^−1^ and it could reach equilibrium with an adsorption rate (*k*_2_) of 0.16 g mg^−1^ min^−1^. Adsorption of metaldehyde onto PAC with pH_pzc_ of 7.35 was slightly more effective under alkaline conditions. HA could not effectively be removed by the investigated PAC with maximum percentage removal of 50% in 30 days using 30 mg L^−1^ HA solution and 0.25 g of PAC. Furthermore, it could take a very long time to reach equilibrium; presumably more than 30 days. The presence of HA in the binary system did not significantly affect the amount of metaldehyde adsorbed onto the PAC used in this study but it slowed down the adsorption rate of metaldehyde while speeding up the adsorption rate of HA. This could be explained by the fact that small metaldehyde molecules would prefer the abundant micropores and mesopores of PAC while large and complex HA would only attach to the surface of PAC or adsorbed onto the less common macropores of PAC. When two compounds appear in the same adsorption system, the system would seek to balance the adsorption rate of each compound. Adsorption of metaldehyde onto the PAC used in this study could be best described by the Langmuir isotherm model and pseudo-second order model, suggesting this adsorption process can be explained by the attachment of a single layer of metaldehyde molecule onto the surface of PAC and promoted by intraparticle diffusion. Understanding the adsorption mechanism of metaldehyde by PAC contributes to enhancing the application of PAC in water treatment plants. Since the presence of HA showed not affect the removal of metaldehyde in the binary system by PAC, there are many water treatment stages to choose from such as coagulation/flocculation and pre-ozonation to add PAC in. PAC could then be removed from a later stage such as sedimentation or filtration.

Some aspects of this research are recommended to be further studied. The mechanism of interaction of pollutants with the surface groups of activated carbon is a complex phenomenon, especially when working with activated carbons with heterogeneous surface, as is the case of this work. The interactions depend on the nature of the contaminant (metaldehyde), and on the surface groups, as well as the state of their ionization, related with the pH of the medium. For that reason, the studies to explain the mechanisms of action of metaldehyde with surface groups are ongoing at this moment and the results will be published soon. In addition, possible desorption of metaldehyde from PAC back into water will be studied along with possible regeneration of PAC to investigate the number of cycles that PAC could be used before becoming exhausted. Moreover, natural water will be used instead of synthetic water in the next step of our research. Metaldehyde solution will be prepared using water from different stages at water treatment plant and the best stage to add PAC in the treatment process will be identified.

## Conflicts of interest

There are no conflicts to declare.

## Supplementary Material

RA-009-C8RA06802J-s001
