# Supplementary material for: The impact of humic acid on metaldehyde adsorption onto powdered activated carbon in aqueous solution
Source: RSC Adv. 2018 Dec 19;9(1):11–22. doi: 10.1039/c8ra06802j (PMC9059139; doi:10.1039/c8ra06802j)
Supplement: RA-009-C8RA06802J-s001 [file RA-009-C8RA06802J-s001.pdf]

Electronic Supplementary Material (ESI) for RSC Advances.
This journal is © The Royal Society of Chemistry 2018

Article

The impact of humic acid on metaldehyde adsorption onto powdered activated carbon in aqueous solution†

Journal name: RSC Advances

Zhuojun Li[a], Yuchen Yang[b], Ulises Jáuregui-Haza[c], Zhengxiao Guo[b], Luiza Cintra Campos[a*]

[a] Department of Civil, Environmental and Geomatic Engineering, University College London, Gower Street, London WC1E 6BT, UK; Emails: zhuojun.li.09@ucl.ac.uk; l.campos@ucl.ac.uk
[b] Department of Chemistry, University College London, Gower Street, London WC1E 6BT, UK; Email: yu.yang.13@ucl.ac.uk; x.guo@ucl.ac.uk
[c] Instituto Superior de Tecnologías y Ciencias Aplicadas (InSTEC), Universidad de La Habana, La Habana, CP 10600, Cuba; Email: ulises.jauregui@infomed.sld.cu

* Author to whom correspondence should be addressed; Email: l.campos@ucl.ac.uk (L.C.C.); Tel.: +44-207-679-4162 (L.C.C)

† Electronic supplementary information (ESI) available.

Adsorption experiment:

For single adsorption of metaldehyde, three experiments were done: 1) PAC dosage was varied from 0.005g to 0.5 g using 500 mL of 1 mg L^{-1} metaldehyde solution without adjusting the pH of the solution for a 2-hour reaction time; 2) sample solutions were taken at different time intervals between 0 minutes and 2 hours using 500 mL of 1 mg L^{-1} metaldehyde solution and 0.05 g PAC without adjusting the pH of the solution; 3) pH of metaldehyde solution was varied from 4 to 12 using 500 mL of metaldehyde solution and 0.05 g PAC for a 2-hour reaction time.

For single adsorption of HA, two experiments were done: 1) PAC dosage was varied from 0.05 g to 1 g using 500 mL of 30 mg L^{-1} HA solution without adjusting the pH of the solution for the 2-hour reaction time; 2) the sample solutions were taken at different time intervals between 0 minutes and 30 days using 500 mL of 30 mg L^{-1} HA solution without adjusting the pH of the solution and 0.25 g PAC.

For binary adsorption of metaldehyde and HA, two experiments were done: 1) for the 500 mL multi-component solution containing metaldehyde and HA, the concentration of HA in the binary system was varied from 3 mg L^{-1} to 90 mg L^{-1} and the concentration of metaldehyde was fixed at 1 mg L^{-1} without adjusting the pH of the solution using 0.05 g PAC for a 2-hour reaction time; 2) sample solutions were taken at different time intervals between 0 minutes to 2 hours using 500 mL of multi-component solution containing 1 mg L^{-1} metaldehyde and 30 mg L^{-1} HA without adjusting the pH of the solution and 0.05 g PAC.

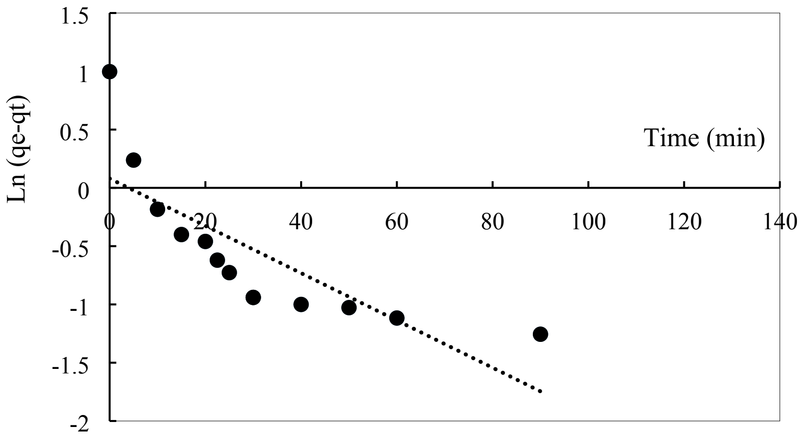

Figure 1. Pseudo-first order kinetic model fitting of 1 mg L^{-1} metaldehyde with 0.05g PAC in 2-hour batch experiment in single system

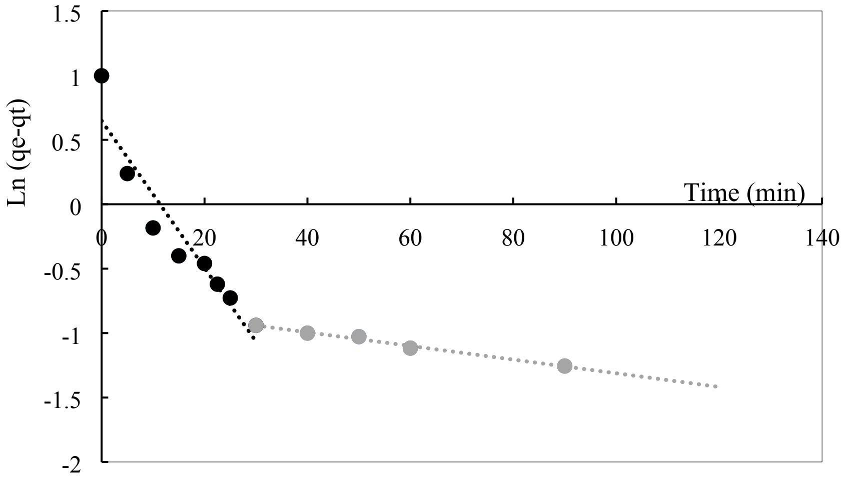

Figure 2. Two gradients plotting of pseudo-first order kinetic model

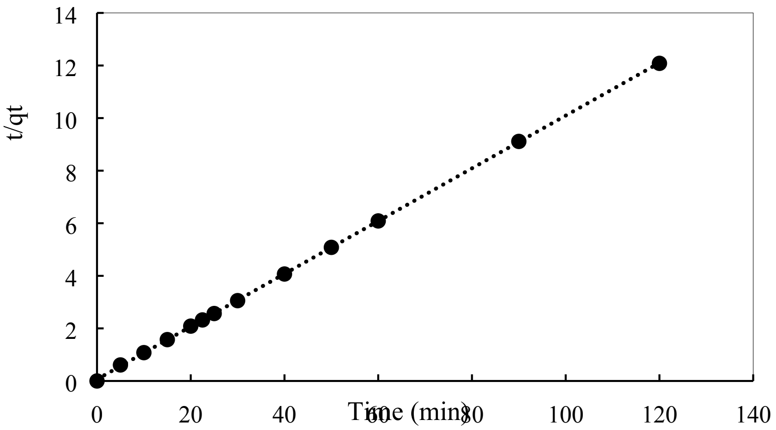

Figure 3. Pseudo-second order kinetic model fitting of 1 mg L^{-1} metaldehyde with 0.05g PAC in 2-hour batch experiment in single system

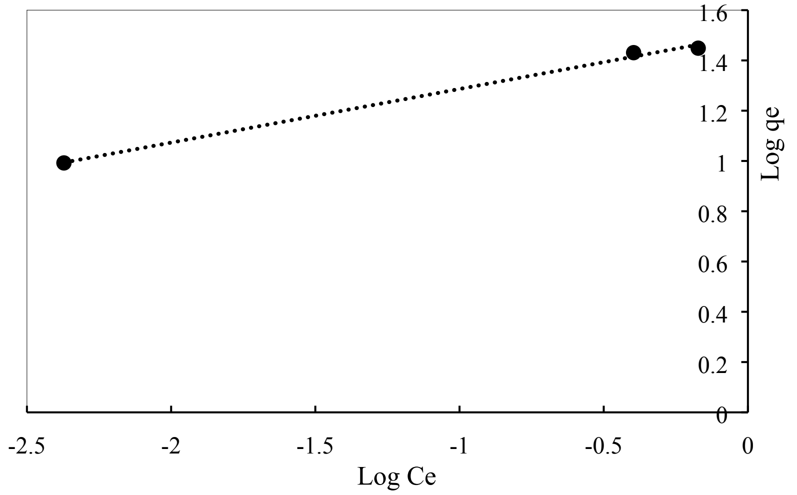

Figure 4. Freundlich isotherm fitting for single adsorption of metaldehyde

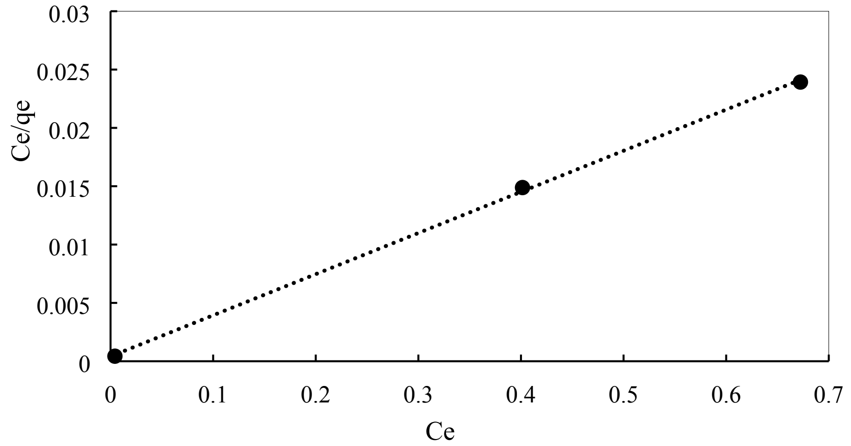

Figure 5. Langmuir isotherm fitting for single adsorption of metaldehyde

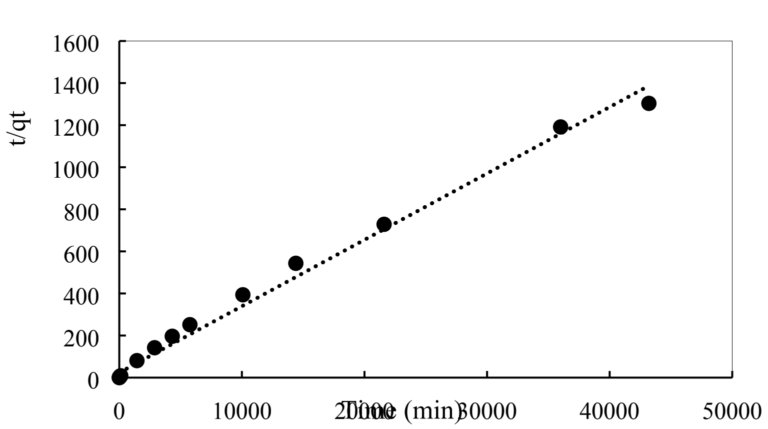

Figure 6. Pseudo-second order kinetic fitting of 30 mg L^{-1} metaldehyde with 0.25g PAC in 2-hour batch experiment in single system

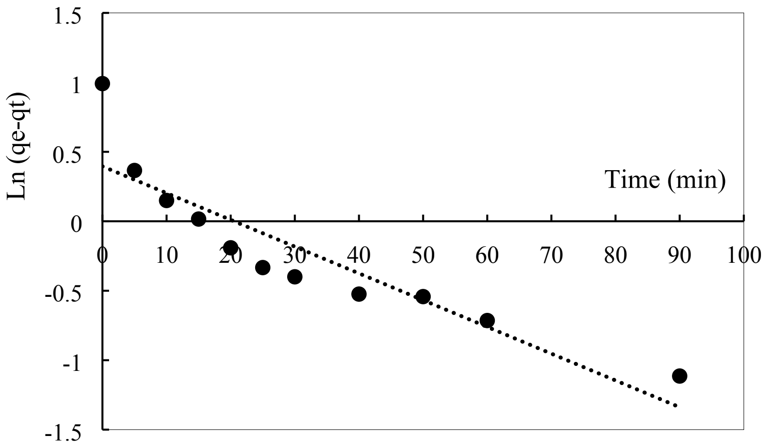

Figure 7. Pseudo-first order kinetic model fitting for metaldehyde in binary system (1 mg L^{-1} metaldehyde and 30 mg L^{-1} HA) with 0.05g PAC in 2-hour batch experiment

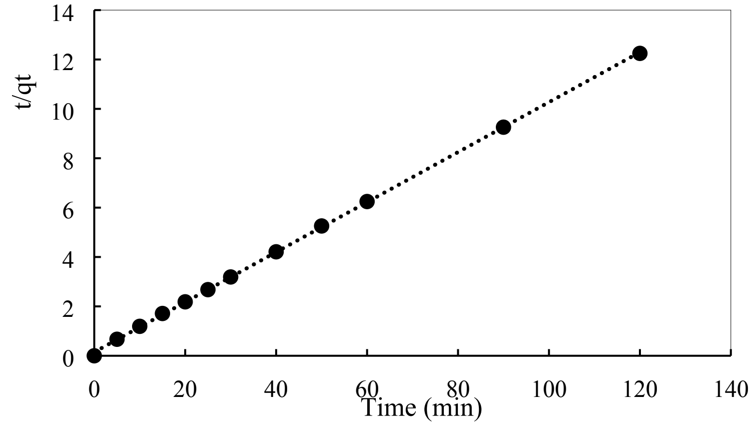

Figure 8. Pseudo-second order kinetic model fitting for metaldehyde in binary system (1 mg L^{-1} metaldehyde and 30 mg L^{-1} HA) with 0.05g PAC in 2-hour batch experiment

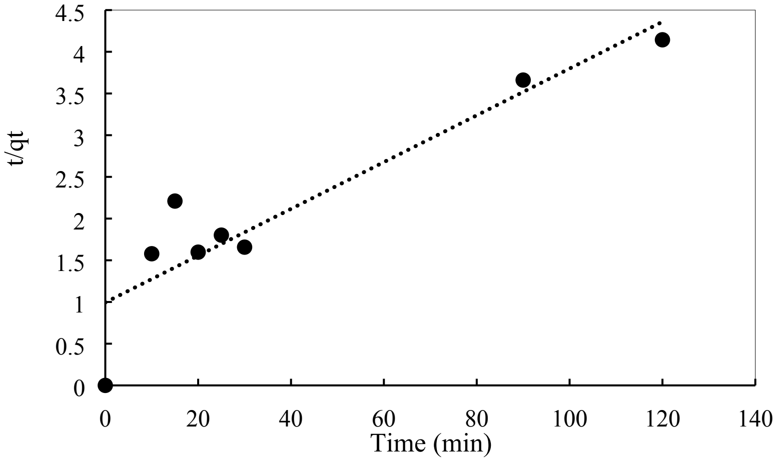

Figure 9. Pseudo-second order kinetic model fitting for HA of in binary system (1 mg L^{-1} metaldehyde and 30 mg L^{-1} HA) with 0.05g PAC in 2-hour batch experiment

Table 1. Removal of metaldehyde and HA under different experiment conditions (p values are from ANOVA single-factor statistic test for analysing concentrations of solutions before and after treatments)

	Percentage removal (%)	Initial concentration (mg L^{-1})		PAC dosage (g L^{-1})	p value
		Metaldehyde	HA		
Removal of metaldehyde (single)	30.3 ± 6.4	1	n/a	0.01	2.28×10^{-7}
	57.8 ± 2.7	1		0.02	1.07×10^{-9}
	99.6 ± 0.0	1		0.1	4.07×10^{-12}
	100.0 ± 0.0	1		0.2	8.24×10^{-16}
	100.0 ± 0.0	1		1	1.45×10^{-12}
Removal of HA (single)	9.8 ± 0.1	n/a	30	0.1	8.5×10^{-10}
	14.8 ± 0.1		30	0.2	6.96×10^{-11}
	21.6 ± 0		30	0.5	6.72×10^{-11}
	25.1 ± 0.1		30	1	1.39×10^{-12}
	32.0 ± 0.1		30	2	9.8×10^{-11}
Removal of metaldehyde (binary)	98.6 ± 0.2	1	3	0.1	1.24×10^{-18}
	98.4 ± 0.3	1	9	0.1	8.48×10^{-16}
	97.8 ± 0.5	1	15	0.1	2.53×10^{-18}
	96.2 ± 0.5	1	30	0.1	1.37×10^{-18}
	89.9 ± 1.4	1	60	0.1	9.76×10^{-16}
	90.2 ± 1.0	1	90	0.1	1.31×10^{-14}
Removal of HA (binary)	20.5 ± 0.5	1	3	0.1	1.98×10^{-7}
	16.3 ± 0.4	1	9	0.1	2.71×10^{-7}
	14.8 ± 0	1	15	0.1	6.53×10^{-11}
	11.3 ± 1.6	1	30	0.1	1.11×10^{-10}
	7.7 ± 0	1	60	0.1	1.34×10^{-10}
	6.5 ± 0	1	90	0.1	8.73×10^{-10}

Table 2. Comparison table of adsorption of metaldehyde and HA onto GAC and PAC

**Sample solution (500 mL)**	**2 g/L GAC (purchased from sigma-aldrich, 20 mesh size, specific surface area = 650 m^2 g^{-1})**		**0.1 g/L PAC (used in this study, specific surface area = 962 m^2 g^{-1})**		**0.2 g/L PAC (used in this study, specific surface area = 962 m^2 g^{-1})**	
	**Removal (%)**		**Removal (%)**		**Removal (%)**	
	**Metaldehyde**	**HA**	**Metaldehyde**	**HA**	**Metaldehyde**	**HA**
**Mono-component solution of metaldehyde (1 mg L^{-1})**	99.70±0.15		99.6		100	
**Multi-component solution of metaldehyde (1 mg L^{-1}) and HA (30 mg L^{-1})**	98.99±0.58	21.56	96.2±0.5	11.3 ± 1.6		
	k_2 = 0.016 g mg^{-1} min^{-1}		k_2 = 0.069 g mg^{-1} min^{-1}			
**Mono-component solution of HA (30 mg L^{-1})**		22.67		9.8 ± 0.1		14.8 ± 0.1
